# Isolated Small Finger Distal Interphalangeal Joint Dupuytren's Contracture

**DOI:** 10.1155/2019/7183739

**Published:** 2019-11-04

**Authors:** Syed K. Mehdi, John D. King, Sara Keshtvarz, Srinath Kamineni, Vikas Dhawan

**Affiliations:** ^1^University of Kentucky Department of Orthopedic Surgery and Sports Medicine, Lexington, Kentucky, USA; ^2^University of Kentucky College of Medicine, Lexington Kentucky, USA

## Abstract

Dupuytren's contracture is a disease involving abnormal myofibroblast proliferation and collagen deposition leading to the formation of pathologic cords in the hand. Given that Dupuytren's contractures rarely extend to the distal interphalangeal joint (DIP), affecting only 5% of patients, there are few cases reported in the literature. Collagenase injection is a frequently used option for minimally invasive treatment of Dupuytren's disease with greater than a 20-degree joint contracture. Unfortunately, there is limited research on the effectiveness of these injections in isolated DIP joint deformities. We present a case of a 61-year-old right hand-dominant male with a 2-year history of isolated right small finger Dupuytren's contracture at the DIP joint who achieved significant improvement after collagenase injection.

## 1. Introduction

Dupuytren's contracture is a disease involving abnormal myofibroblast proliferation and collagen deposition leading to the formation of pathologic cords [1]. These pretendinous cords are formed from pretendinous bands, and they are responsible for the deformation of the plantar fascia and metacarpophalangeal (MCP) joint contractures affecting the palmar, palmodigital, and digital regions of the hand [2]. These bands often extend distally in continuity with digital cords. The most involved digital cords in the pathophysiology of Dupuytren's contracture include the central, spiral, and lateral cord [3]. The central cord is an extension of the pretendinous cord and attaches to the base of the middle phalanx. It does not lead to displacement of the neurovascular bundle but does lead to PIP joint flexion contractures [2, 4]. The spiral cord is comprised of four structures, the pretendinous band, spiral band, lateral digital sheet/band, and the Grayson ligament. This cord is superficial to the neurovascular bundle until is passes the MCP joint, at which point it passes deep to the bundle and progresses laterally and distally [2]. As the disease progresses, the cord straightens causing the neurovascular bundle to displace superficially and towards midline [2]. Finally, the lateral cord may contribute to PIP contracture as well as DIP joint contracture. Combined with the retrovascular cord, these two cords are believed to be responsible for DIP joint contractures [2]. Because Dupuytren's contractures rarely extend to the distal interphalangeal joint, there are few cases reported in the literature as it is believed to affect only 5% of patients [5]. This case report documents a patient with Dupuytren's contracture isolated to the DIP joint of the right small finger.

## 2. Case Report

We present a 61-year-old right hand-dominant male who complained of a 2-year history of right small finger deformity at the DIP joint. He denied any antecedent trauma and was not suffering any numbness or paresthesia in the digit. On exam, there was a nontender palpable nodule on the radial aspect of the right small finger DIP joint. He had an approximately 80° flexion deformity at rest which was correctable to 55° flexion with passive motion. Based on the original Tubiana staging system, this patient had stage 2 disease with a flexion deformity between 45° and 90° [6]. Because this deformity was regularly affecting his activities of daily living such as buttoning shirts, typing on the computer, and writing with a pen, we discussed the benefits and risks of various treatment options, including the more traditional surgical fasciotomy versus a collagenase *Clostridium histolyticum* (CCH) injection [5, 7]. Other important social factors of the patient include that he was employed as a professor and was a nonsmoker. The patient chose to proceed with a collagenase injection.

**Figure 1:**
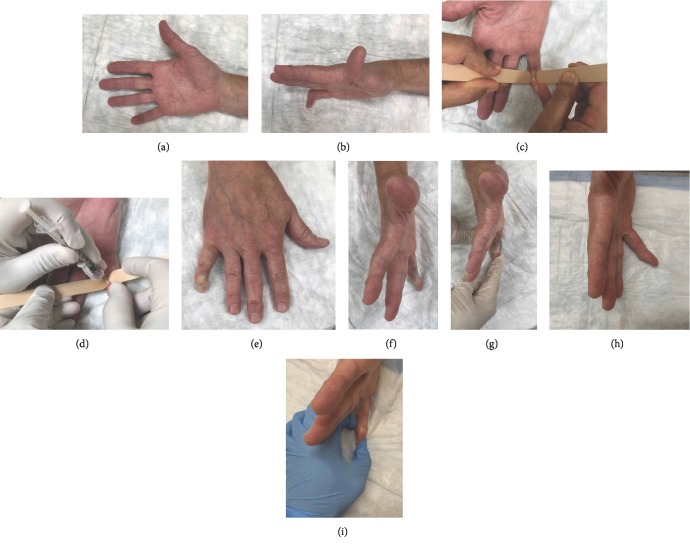
Dupuytren's contracture cord of the DIP joint (a, b). Injection of Dupuytren's contracture cord (c, d). Postinjection results (e–g). Postinjection results at 5 months (h, i).

Prior to performing the procedure, the patient was reexamined to verify that Dupuytren's contracture with a palpable cord was present and informed consent was obtained. Risk and benefits to the procedure were discussed which included injury to tendons, infection, nerve injury, or an allergic reaction to the injection. Once the patient understood and agreed to proceed, the area to be injected was prepared with a betadine solution, and using a sterile glove, the cord was palpated. An assistant then placed a tongue depressor on both sides of the cord (see Figure 1), in order to isolate the cord from the surrounding tissue, stabilize it, and bring it into greater visualization, per the senior authors technique. Collagenase (0.58 mg) was then injected into the cord according to manufacturer's recommendations, Endo Pharmaceuticals, Malvern, PA. Because the tongue depressors continually isolated and stabilized the cord during the injection, the physician was able to keep his second hand on the barrel of the needle to provide counterpressure and prevent migration of the needle tip out of appropriate position while administering the injection. A soft dressing was applied after the injection, and the patient returned to the clinic in 48 hours for digit manipulation per the senior author's standard practice. He was placed in a dynamic night splint for 4 months as well as prescribed therapy for range of motion to begin immediately to focus on flexion and extension. This protocol is per the Xiaflex injection guide which includes 4 months of night splinting.

At 5 months status post injection, the patient had active range of motion of 10°-80° in the right small finger DIP joint. His joint had 10° of flexion at rest that could be corrected to 0° with the assistance of the other hand (see Figure 1). He expressed improvements in his activities of daily living such as reaching into his pant pockets, typing, and writing with no neurovascular complications seen by the patient or examiner. He denied any pain, loss of sensation in the injected finger, bruising, or loss of function.

## 3. Discussion

Distal interphalangeal joint contractures are quite rare in Dupuytren's disease [8]. A study by Millesi originally described only 5% of patients with Dupuytren's contracture having involvement of the DIP joint with only one case out of 287 patients having isolated DIP contracture [9]. A recent study by Fei et al. described only 2.7% of 775 patients with Dupuytren's contracture having DIP involvement and only 1 patient having an isolated DIP contracture [10]. A successful correction with a CCH injection is defined as being <5° of flexion contracture at 30 days post injection [10, 11]. Historically, collagenase injections have had correction rates of 77% for MCP contractures and 40% for PIP contractures. Fei et al. achieved a correction rate of 84% in 21 patients with DIP contractures, with a mean correction of 26° [10]. Finally, a study by Skov et al. found that Dupuytren's contracture treated with one collagenase injection was equally effective yet cost 33% less than fasciectomy [12]. Given this high correction rate, lower cost, coupled with previous success in our practice with MCP and PIP injections, we were confident in proceeding with a CCH injection for the DIP joint nodule. As successful correction with a CCH injection results in <5° of residual contracture, this patient did not meet these parameters as he had a residual flexion contracture of 10°. One cause of this remaining flexion contracture may be the large degree of initial deformity of 80° and contracture of the collateral ligaments of the DIP joint. Fei et al. had an 84% success rate of DIP contracture correction but their mean correction was only 26° [10]. Similarly, a case series of 9 patients by Denkler had a 66% success rate of DIP contracture correction with a mean correction of only 12° [13]. Unlike other studies, this patient underwent a large correction of 70°. Nevertheless, he was very pleased with his outcome as he had significant improvement in his activities of daily living. Incomplete deformity correction and reoccurrence, defined as more than 20° of contracture recurrence in any treated joint at one year post treatment [14], are potential risks of CCH injections. Previous studies have demonstrated a reoccurrence rate ranging from as low as 12% to as high as 83% [7, 12, 15, 16]. As a result, it is important to discuss the risk of incomplete correction and reoccurrence with patients prior to proceeding with an injection. In this case report, however, our patient had significant correction of a rare isolated DIP joint Dupuytren's contracture after CCH injection and was pleased with the clinical results.
